# Supplementary material for: Single-cell RNA sequencing reveals differential cell cycle activity in key cell populations during nephrogenesis
Source: Sci Rep. 2021 Nov 17;11:22434. doi: 10.1038/s41598-021-01790-6 (PMC8599654; doi:10.1038/s41598-021-01790-6)

Supplemental Information for the manuscript

Single cell RNA sequencing reveals differential cell cycle activity in key cell populations during nephrogenesis

Abha S. Bais[1+], Débora M. Cerqueira[2,3+], Andrew Clugston[1,2,3+], Andrew J. Bodnar[2,3]

Jacqueline Ho[2,3*] and Dennis Kostka[1,4*]

[1]Department of Developmental Biology, University of Pittsburgh School of Medicine, Pittsburgh, PA, USA

[2]Rangos Research Center, UPMC Children's Hospital of Pittsburgh, Pittsburgh, PA, USA

[3]Department of Pediatrics, Division of Nephrology, University of Pittsburgh School of Medicine, PA, USA

[4]Department of Computational & Systems Biology and Pittsburgh Center for Evolutionary Biology and Medicine, University of Pittsburgh School of Medicine, Pittsburgh, PA, USA

[+]These authors contributed equally to this work.

[*]Co-Corresponding authors:

Dr. Dennis Kostka
Rangos Research Center 8117
Department of Developmental Biology
530 45th St
Pittsburgh, Pennsylvania 15224
USA
Phone: 412-692-9905
Email: kostka@pitt.edu

Dr. Jacqueline Ho
Rangos Research Center 5127
Department of Pediatrics
530 45th St
Pittsburgh, Pennsylvania 15224
USA
Phone: 412-692-5303
Email: jacqueline.ho2@chp.edu

SUPPLEMENTAL TABLE LEGENDS

Supplemental Table S1: ***Marker genes that distinguish between kidney cell types.*** For each cluster marker genes with an FDR < 0.05 are reported (see **Fig. 1**).

Supplemental Table S2: ***Marker genes that distinguish nephron-progenitor derived cell types.*** For each cluster (imperfect) marker genes with an FDR < 0.05 are reported (see **Fig. 2**).

Supplemental Table S3: ***Differentially expressed genes for self-renewing vs. primed nephron progenitor cell types.*** For each comparison, differentially expressed genes (FDR < 0.1) are reported (see **Fig. 3a**).

Supplemental Table S4: ***Differentially expressed genes for primed nephron progenitor cells vs. differentiating cells.*** Differentially expressed genes (FDR < 0.1) are reported (see **Fig. 3a**).

Supplemental Table S5: ***Gene Ontology terms enriched for differentially expressed genes, comparing self-renewing vs. primed nephron progenitor cells and primed vs. differentiating cells.*** See **Fig. 3b and 3c**.

Supplemental Table S6: ***Genes associated with pseudotime across nephron progenitor cells.*** Genes associated with pseudotime are reported (FWER < 1%).

Supplemental Table S7: ***Gene sets enriched for genes increasing with pseudotime and for genes decreasing with pseudotime across nephron progenitor cells.*** Gene sets from MSigDB for Gene Ontology (Biological Process) and Hallmark gene sets (FDR < 0.01) are reported.

Supplemental Table S8: ***Regulatory modules.*** Regulatory modules of genes active in the transition between self-renewing and primed nephron progenitor cells (see **Fig. 3g**).

Supplemental Table S9: ***Differentially expressed genes for mature vs. immature nephron progenitor derived cell types.*** Differentially expressed genes (FDR < 0.1) are reported (see **Fig. S5**).

Supplemental Table S10: ***Differentially expressed genes for distal vs. proximal tubular cells.*** Differentially expressed genes (FDR < 0.1) are reported (see **Fig. S5**).

Supplemental Table S11: ***Differentially expressed genes for self-renewing vs. primed nephron progenitor cell types after removing cell cycle-related genes.*** Differentially expressed genes (FDR < 0.1, log fold change cutoff of 1.2) are reported. Genes with percentage of variance explained by cell cycle phase >5% were excluded (code available on GitHub repository).

Supplemental Table S12: ***Marker genes for self-renewing vs. primed nephron progenitor cell types after regressing out cell cycle scores.*** For each cell type marker genes with an FDR < 0.05 (log fold change cutoff of 1.2) are reported (code available on GitHub repository).

Supplemental Figure S1

Heatmap of marker genes that distinguish between kidney cell types. Rows are genes (fifteen top-most marker genes have been selected for each cluster), and columns are cells grouped by cell types.

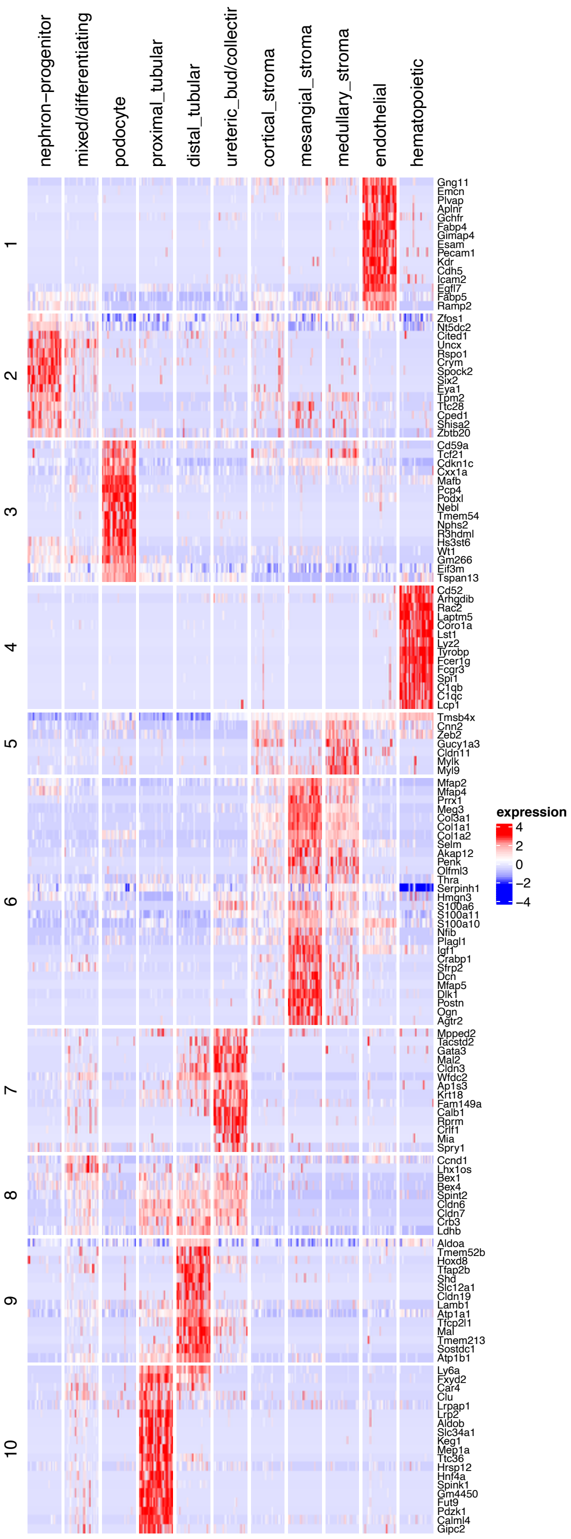

Supplemental Figure S2

Differentiation lineages for nephron progenitor cells. A tSNE embedding for nephron progenitor cells is shown. Differentiation lineages inferred by the slingshot R package are displayed in gray.

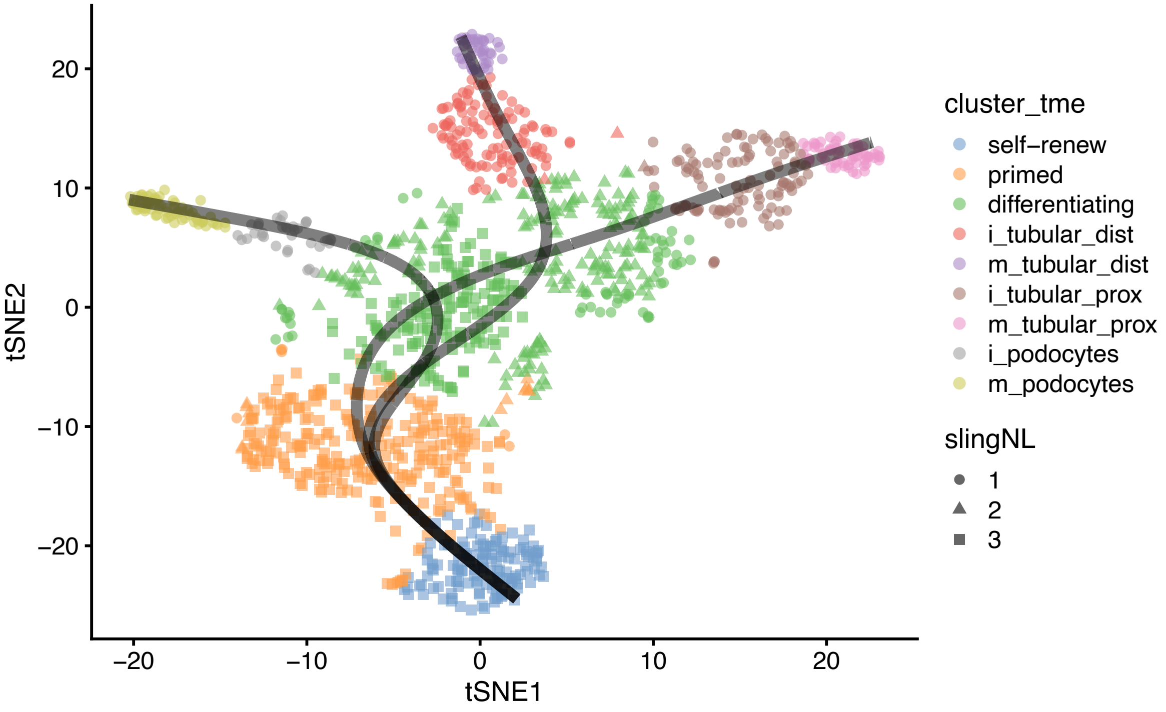

Supplemental Figure S3

Differentially expressed genes during nephron progenitor cell differentiation.
Heatmap of top differentially expressed genes between annotated clusters (column labels).

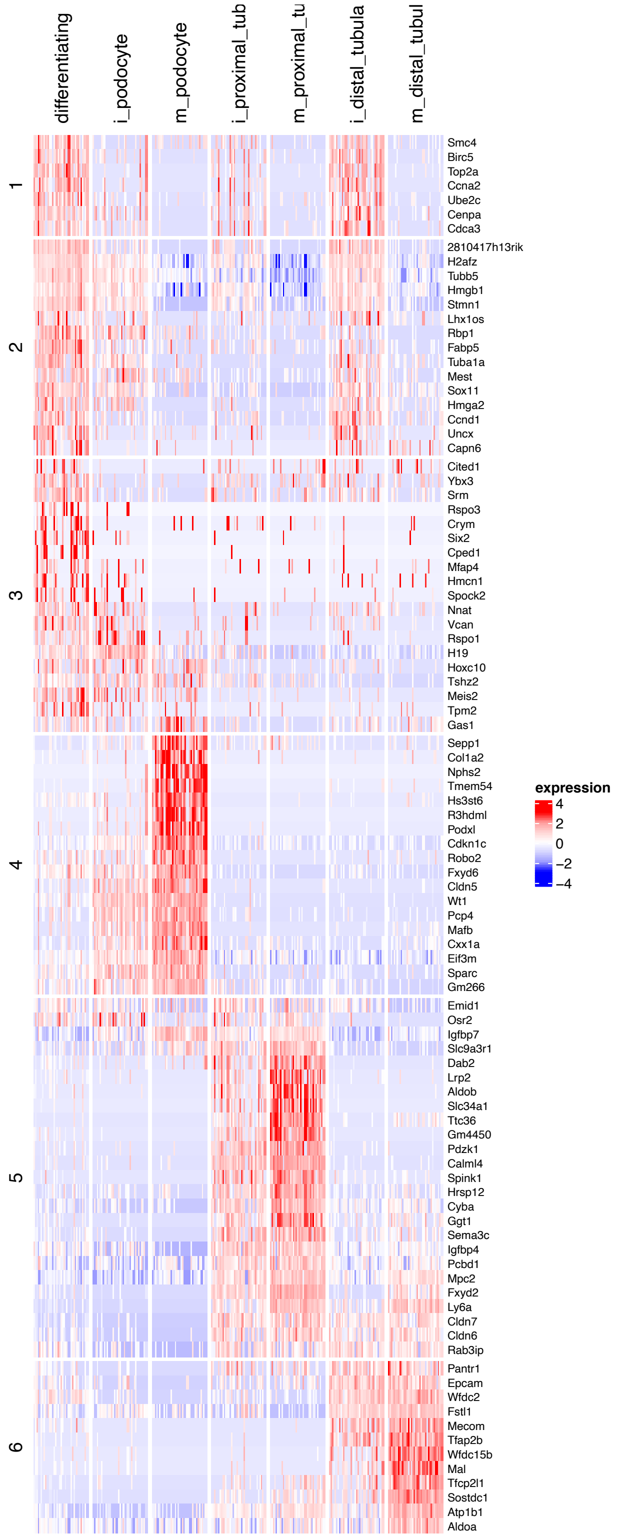

Supplemental Figure S4

Birc5 expression, stratified by annotated cell-cycle phase.

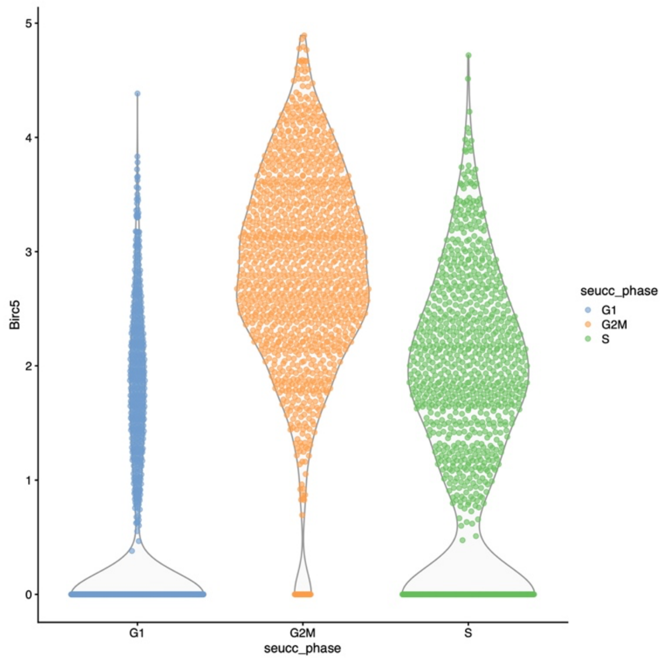

Supplemental Figure S5

This figure shows NP and NP-derived cells together with their cell type annotations (top left), and the same annotations after regressing out cell-cycle (top right). The bottom row shows cell cycle scores for G2/M and S scores and annotated phase for each cell using the tSNE projection of the top-left panel. We used Seurat [PMID: 34062119; 10.1016/j.cell.2021.04.048] for cell cycle phase scoring. (code available on GitHub repository).

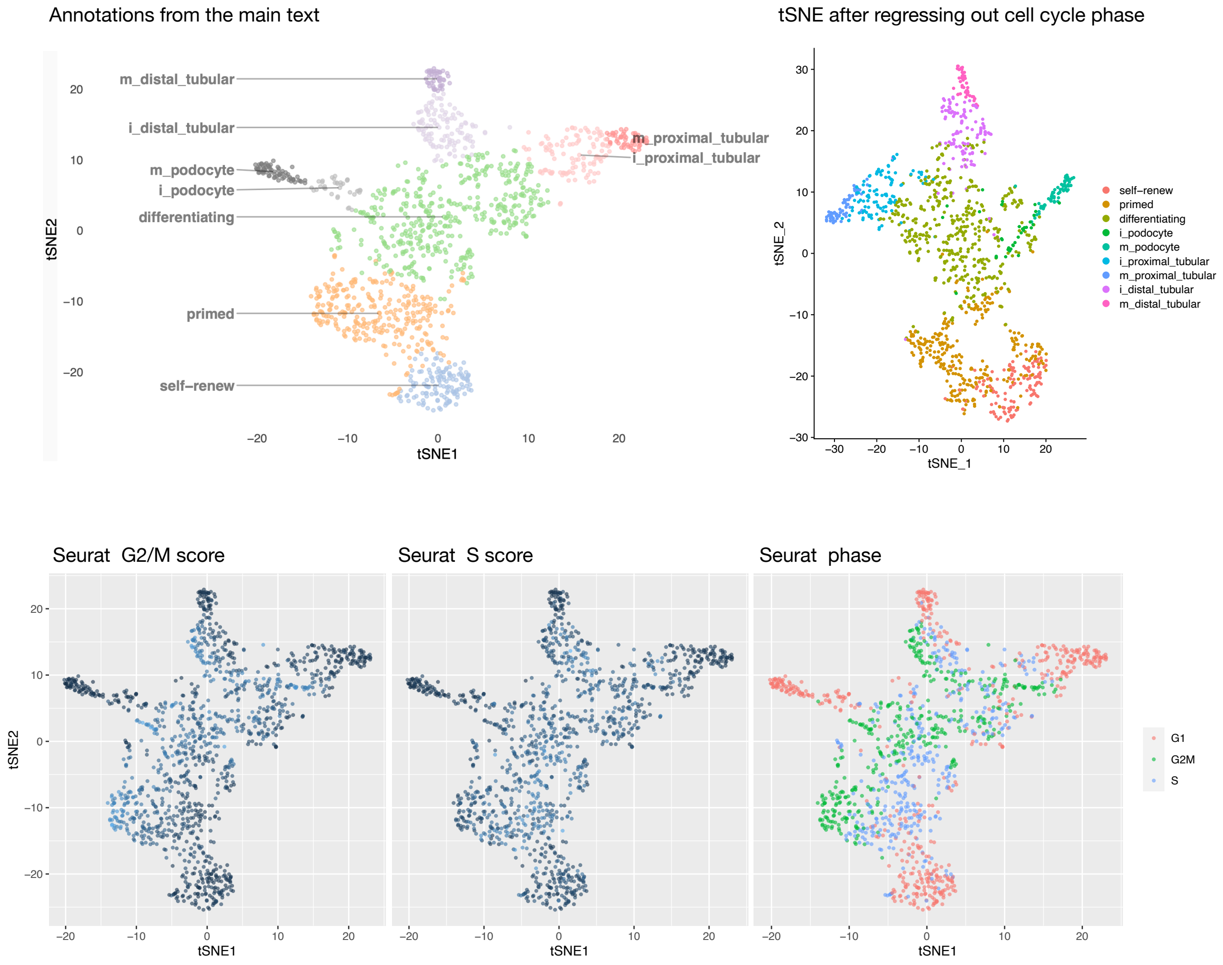

Supplemental Figure S6

Consistent* Birc5 *expression in distal tubular cells and a subpopulation of cells from the ureteric bud. Shown are tSNE plots of nephron progenitor derived cells and cells of the ureteric bud / collecting duct (ub/cd) cluster (see **Figure 1**). ***(a)*** Cell-type annotations for depicted cells. ***(b)*** *Birc5* expression. Arrows denote early distal tubular cells and ub/cd cells with similar *Birc5* expression.

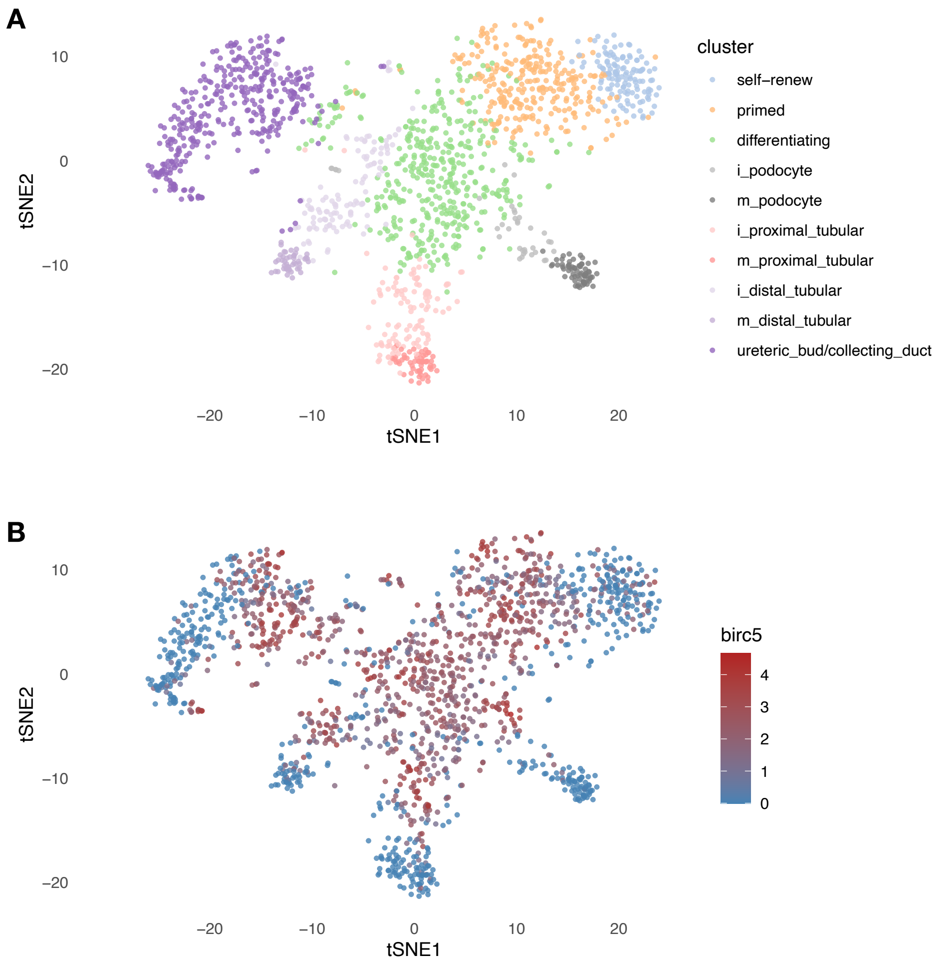

Supplemental Figure S7

Differentially expressed genes in the podocyte and tubular lineages. (a) Heatmap of top 100 differentially expressed genes between immature and mature podocytes and ***(b)*** between proximal and distal tubular cells. ***(c)*** Differential gene expression between immature and mature proximal tubular cells, and ***(d)*** between immature and mature distal tubular cells.

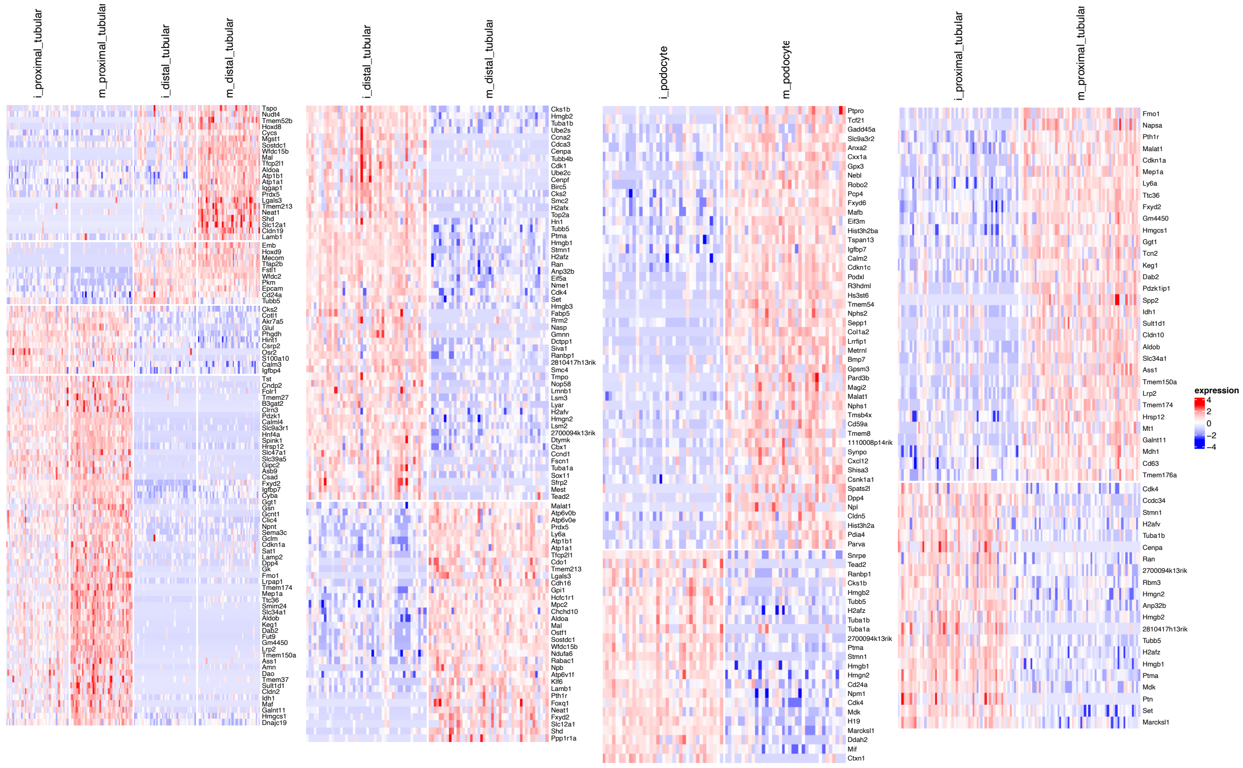

Supplemental Figure S8

Neat1 expression in kidney cells. Cluster annotation and *Neat1* expression across the whole kidney (left, A-C) and in nephron-progenitor and derived cell types (right, D-F). Cluster annotation is the same as in the main text, with the exception that for the whole kidney (A-D) we have used annotation from panel D to differentiate between distal and proximal tubular cells (where we in include both, the mature and immature cells). We see that *Neat1* expression is highest in mature distal tubular cells, and that it is reasonably specific.

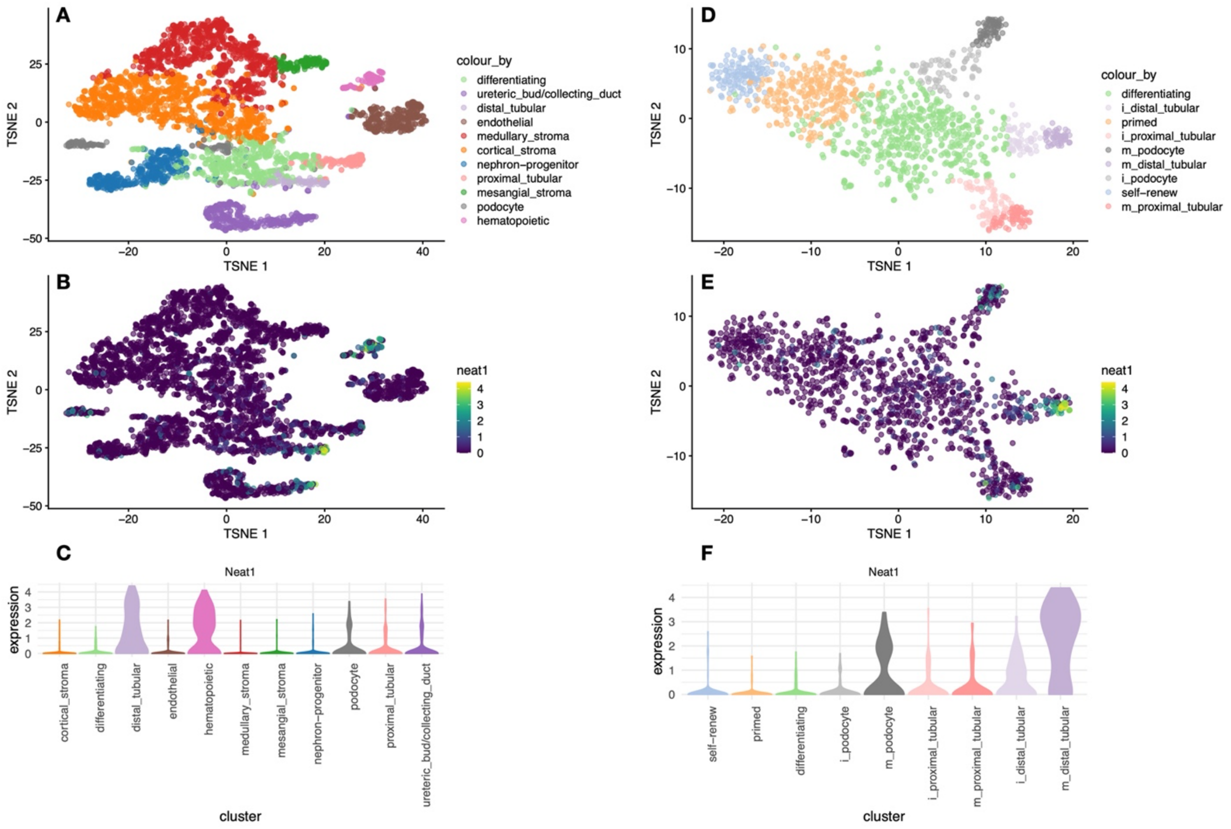

Supplemental Figure S9

Comparison with Magella et al. a) Comparison of cell type annotations in the whole kidney from Magella et al. (ref [27] in the main text) with our annotations that were transferred to this data. Columns are transferred labels, rows are cell type annotations from Magella et al., and color codes for the fraction of cells in each row annotated to each column (i.e., the fraction of cells from each of Magella et al. cell type in each of the transferred cell types). b) tSNE projection of NP and NP-derived cells in the Magella et al. data set. Left panel shows cell types as annotated by Magella et al. Middle panel shows *Birc5* expression and the right panel shows AUCell scores of 'self_renew' NP cells. Shaded circles highlight cap mesenchyme, *Birc5*-expressing and 'self_renew' cells, respectively. (code available on GitHub repository)

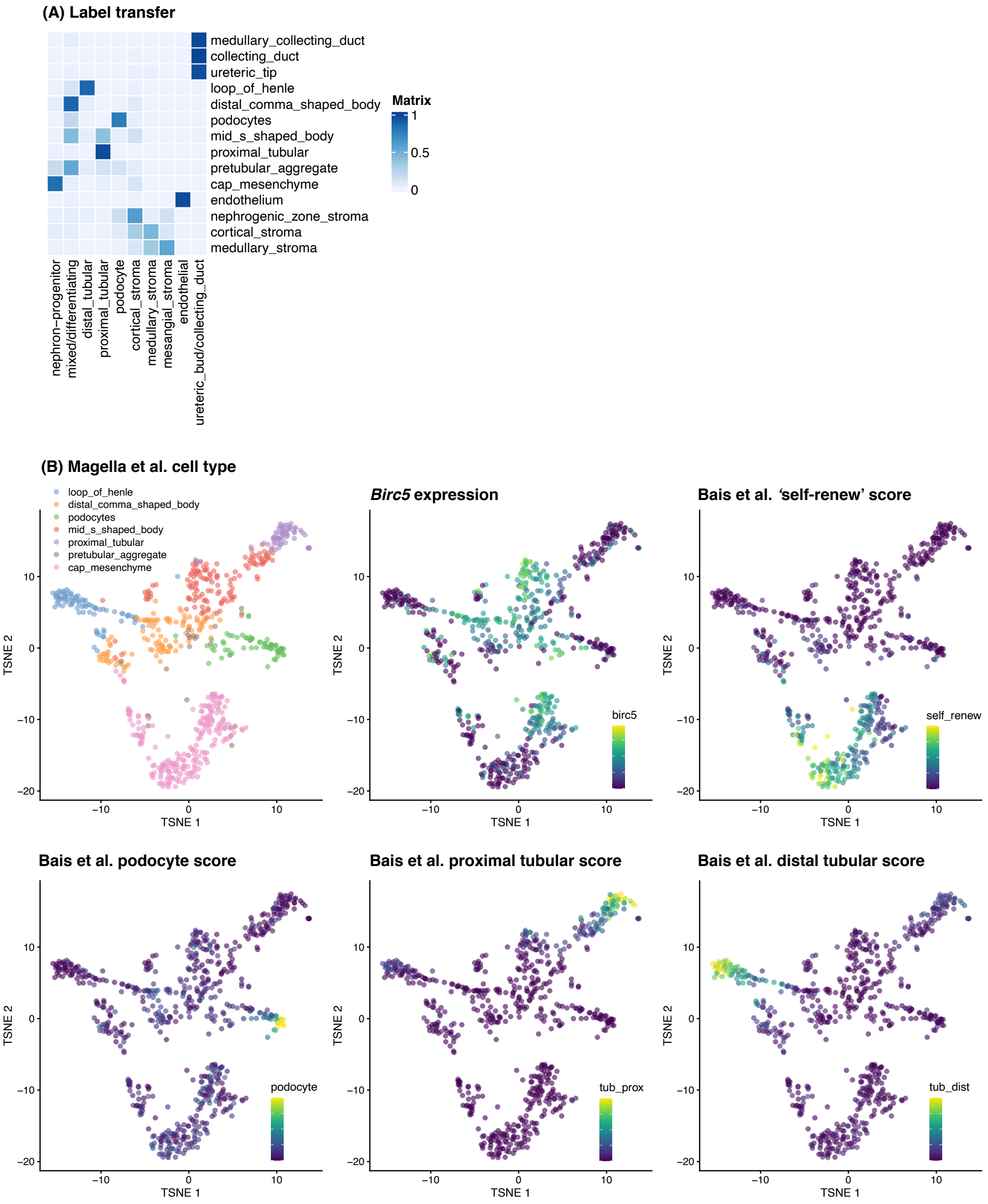

Supplemental Figure S10

Gene expression in 'self-renew' vs. 'primed' NP cells. Genes associated with epithelial differentiation are shown in (A), and genes typically expressed in NP cells are shown in (B). Wilcoxon rank sum test p-values are displayed.

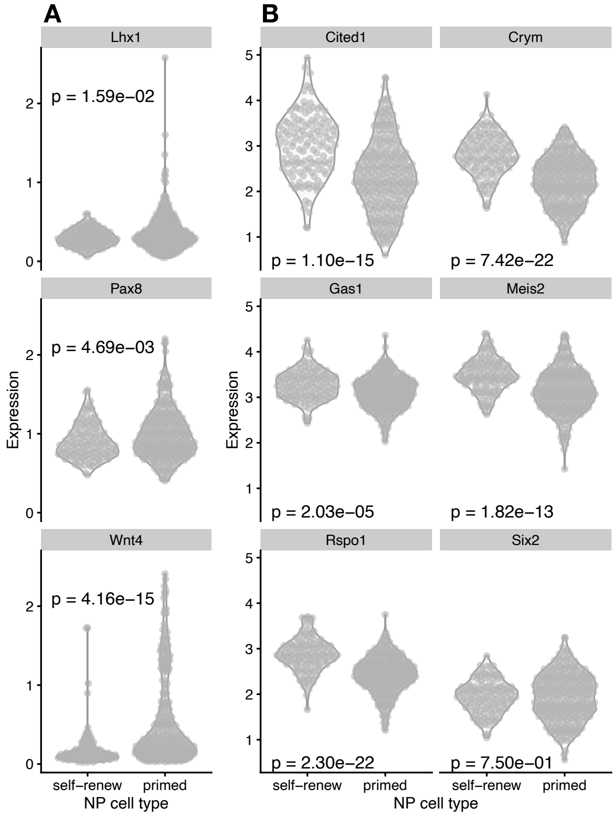

Supplement: Supplementary file 1 — Supplementary Information 1. [file 41598_2021_1790_MOESM1_ESM.pdf]
